# Aging Mechanisms and Non-Destructive Aging Indicators of XLPE/CSPE Unshielded LV Nuclear Power Cables Subjected to Simultaneous Radiation-Mechanical Aging

**DOI:** 10.3390/polym13183033

**Published:** 2021-09-08

**Authors:** Ramy S. A. Afia, Ehtasham Mustafa, Zoltán Ádám Tamus

**Affiliations:** 1 Department of Electrical Power & Machines Engineering, Faculty of Engineering, Helwan University, 1 Sherif Street, Helwan 11792, Egypt; ramysaad@h-eng.helwan.edu.eg; 2 Department of Electric Power Engineering, Faculty of Electrical Engineering & Informatics, Budapest University of Technology & Economics, P.O. Box 91, H-1521 Budapest, Hungary; mustafa.ehtasham@vet.bme.hu; 3 Department of Electrical Engineering, Faculty of Engineering & Technology, Gomal University, Dera Ismail Khan 29050, Pakistan

**Keywords:** low-voltage, nuclear cables, radiation-mechanical aging, degradation mechanisms, dielectric response, extended voltage response, Shore D hardness

## Abstract

Low-voltage cable systems in nuclear power plants are key components that have a crucial role in the safe operation of nuclear facilities. Thus, the aging management of cable systems is of utmost importance as they cannot easily or economically be replaced or upgraded. Therefore, there is a continuous need to develop reliable non-destructive condition monitoring techniques, mostly based on the measurement of the dielectric properties of cable insulation. This paper introduces the changing of dielectric and mechanical properties of XLPE insulated and CSPE jacketed unshielded low-voltage nuclear power plant power cable in case of simultaneous mechanical and radiation aging. The cable samples were bent and exposed to 400 kGy gamma irradiation with a 0.5 kGy/hr dose rate. Dielectric response (real and imaginary permittivity) in the 0.1 Hz−1 kHz frequency range, extended voltage response (EVR), and the Shore D hardness test techniques were measured to track aging. The electrical and mechanical parameters have increased monotonically with aging, except the imaginary permittivity, which increased only at frequencies higher than 10 Hz. Furthermore, different quantities were deducted based on the frequency and permittivity data. The electrical parameters and deducted quantities correlation with aging and mechanical parameters were investigated. Since the deducted quantities and the electrical parameters are strongly correlated with absorbed dose and mechanical properties, the electrical measurements can be applied as a non-destructive aging indicator for XLPE/CSPE unshielded low-voltage nuclear power cables.

## 1. Introduction

Most of the current fleet of nuclear power plants (NPPs) have reached the end or working beyond their initial qualification license to operate for 40 years [1,2]. Currently, the majority of the NPPs have been granted a license renewal to operate for an additional period of 20 years [3]. Furthermore, regulators and plant operators are considering extending the operating license to 80 years [1]. For the safe and reliable operation of an NPP, it is essential to demonstrate that all the plant safety-related elements can fulfill their intended functions under both the normal operation and postulated events such as loss of coolant accident (LOCA) [4,5]. As a result, age-related degradation of NPP components has received more attention from regulators. Hence intensive research work has been done at the national and international level involving governmental, industrial, and academic research laboratories [1,6,7]. 

**Figure 1:**
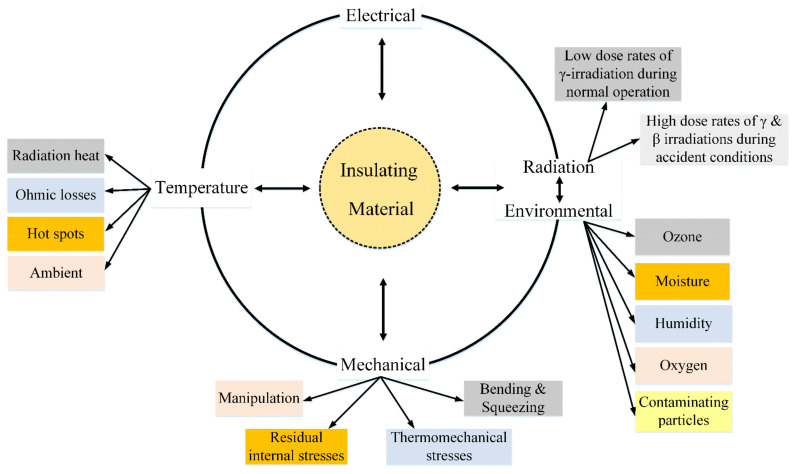
Aging stressors and multi-stress aging.

Cables transmit control signals and data, communicate between different NPP systems, and power various apparatuses [7]. The predominant cable types used in the NPPs are LV power cables and I&C cables since a typical NPP may compose about 1500 km of LV cables [5,8]. NPP LV cables are exposed to harsh conditions during their operation, and they are exposed to different stresses, as illustrated in Figure 1 [9,10]. However, recent studies suggest that heavy metal perovskite materials that absorb high-energy rays can be used to absorb potential hazards [11,12]. The insulation and outer jackets suffer from degradation due to aging stresses, while conductors are less sensitive to aging. However, the jacket material could be a sensitive indicator of aging; the insulation contributes mainly to the electrical and mechanical degradation of cables [13]. Therefore, it is essential to monitor the insulation state to avoid cable failures which may cause catastrophic accidents [14]. 

In addition to the various aging factors, the composition of used jacket and insulating materials also play an important role in aging. The insulation of NPP cables is mainly based on polymers such as cross-linked polyethylene (XLPE), ethylene-propylene rubber (EPR), and chlorosulphonated polyethylene (CSPE) [13,15]. Most of the polymers used in cables contain several additives to enhance the chemical, physical, and electrical properties [16]. These additives include antioxidants, flame retardants, coloring agents, fillers and curing agents, plasticizers, initiators, stabilizers, and other chemicals [17,18]. Additives have a pivotal role in the physicochemical and degradation characteristics of cable materials [15,16,19,20]; therefore, it is crucial that the formulation of the cable material is traceable and documented, particularly for critical applications such as NPPs [21]. 

Extensive research work has been conducted to explore the effect of thermal and radiation stresses on the insulation integrity of NPP cables; however, less attention has been paid to the role of mechanical stresses and their impact on the cables’ performance. Moreover, mechanical aging is not included in the type testing of NPP cables. The simultaneous action of mechanical stresses with ionizing irradiations and elevated temperatures is an essential aging factor that should be considered. Cables’ insulation must withstand the external applied mechanical stresses, for instance, bending during installations [22]. NPP cables’ functionality and integrity are tested by qualification. However, the qualification standards can prescribe mechanical stress (coiling a mandrel) to demonstrate adequate flexibility and lack of embrittlement after radiation and thermal aging, there are no recommendations for the bending or other mechanical loading during the aging procedure [23]. Nevertheless, at some locations in NPPs, the installation bending radius does not fulfill the installation guidelines [17]. Depending on the cable diameter and the bending radius, if the bending radius is sufficiently small, the insulating material is elongated, and cracks are initiated. Thus, the jacket material excessively aged more than the underlying insulation [18].

Conventionally, condition monitoring of NPP cables insulations is based on mechanical testing, particularly the elongation at break (EaB) test with a 50% acceptance criterion. However, the EaB measurement is a commonly used method to determine the degradation of polymers [24,25]. However, the EaB test is naturally destructive, and it requires samples removal; thus, it is not practical for on-site testing [2,5,10,26]. Therefore, non-destructive electrical-based condition monitoring techniques have been the focus of scientific interest, such as dielectric spectroscopy, return voltage measurement, and the polarization-depolarization current method [8,9,10,14,15,19,20,26,27,28,29,30]. These techniques have been successfully adapted to various insulation systems [2,5,26,30]. 

The current work investigates the degradation mechanisms of NPP cables in a multi-stress environment, particularly the simultaneous exposure to radiation and mechanical stresses where the aging process becomes more complex. The assessment of the insulation condition was based on electrical and mechanical non-destructive techniques. In particular, the real and imaginary parts of permittivity, the extended voltage response, and the Shore D hardness as a mechanical property were tested. For quantitative evaluation of dielectric spectrum, deducted quantities were introduced, and correlation between mechanical and dielectric properties was also investigated.

## 2. Research Approach

**Figure 2:**
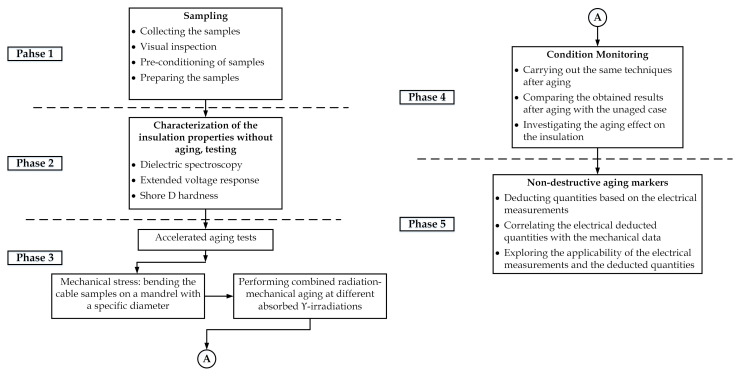
Flowchart of the research approach.

The research approach is based on an accelerated aging test to simulate stresses in operation and the investigation of changing the electrical and mechanical properties due to aging. The research approach is illustrated in Figure 2. The entire work involves five phases. The cable samples have been selected, visually inspected, prepared, and pre-conditioned, in the first phase. While in the second phase, the measurement of electrical and mechanical properties was carried out on unaged samples. The accelerated aging was performed in the third phase. In the fourth phase, the cable samples were measured again to investigate the impact of aging. Seeking for the so-called non-destructive aging indicators, deducted quantities based on electrical measurements, particularly frequency domain data, were calculated in the last phase. Moreover, the implementation of the deducted quantities was investigated via correlation with the absorbed dose and mechanical parameter, hardness.

## 3. Materials and Samples Preparation

**Figure 3:**
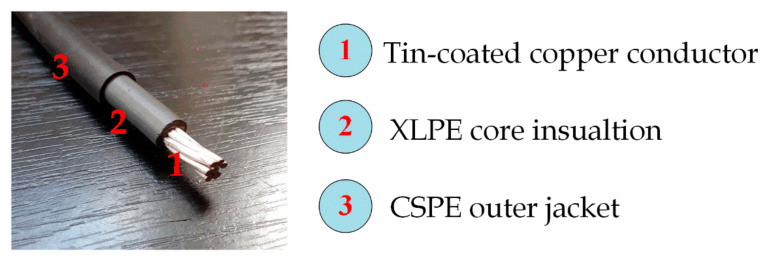
The cable under investigation.

The entire work has been carried out on Firewall III-J radiation-resistant class 1E LV unshielded nuclear power cable (RSCC Wire and Cable, East Granby, CT, USA). This cable type is qualified with a minimum of 40-years thermal life at 90 °C. As illustrated in Figure 3, the NPP cable comprises three parts: annealed tin-coated copper conductor, primary insulation of XLPE, and outer jacketing of CSPE. 

The cable technical data are listed in Table 1.

**Figure 4:**
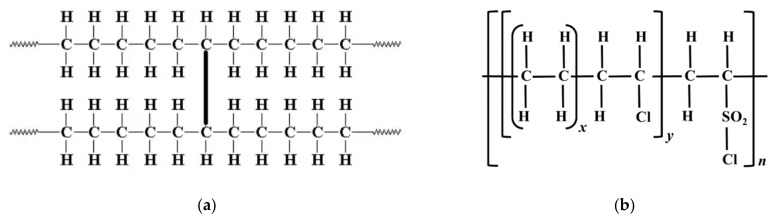
Chemical illustration of XLPE (**a**) and CSPE (**b**).

Figure 4 illustrates the chemical structure of both XLPE (a) and CSPE (b). XLPE is a form of polyethylene (PE) with cross-linked bonds. Due to the cross-linking process, the XLPE possesses leading properties than the PE [31]. The cross-linking can be achieved via three different cross-linking types: silane cross-linking, peroxide cross-linking, and radiation cross-linking. The CSPE is obtained through the coinciding chlorination and chlorosulfonation of PE. CSPE is a polymer that involves a modified PE backbone with chloro and sulfonyl chloride side groups. Furthermore, cross-linking can be achieved with different curing methods [32].

**Figure 5:**
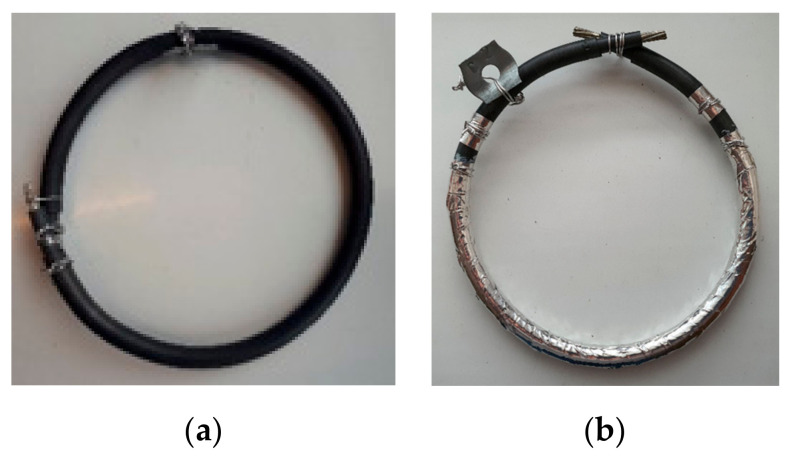
A prepared cable sample for (**a**) simultaneous radiation-mechanical aging, and (**b**) electrical measurements by wrapping foil electrode to the outer surface.

The samples’ length was 50 cm. According to the IAEA guidelines [7], 1 and 3 cm have been peeled from the core insulation and jacket to avoid leakage current during the measurements. As already stated, the NPP cable samples were exposed to simultaneous radiation-mechanical aging. The mechanical stress for aging was induced by the bending of the cable. The samples have been coiled on a mandrel and fixed tightly at both ends, as demonstrated in Figure 5a. The samples were removed from the cylinder, keeping the bending radius constant, which was 2.75 inches according to the manufacturer.

**Figure 6:**
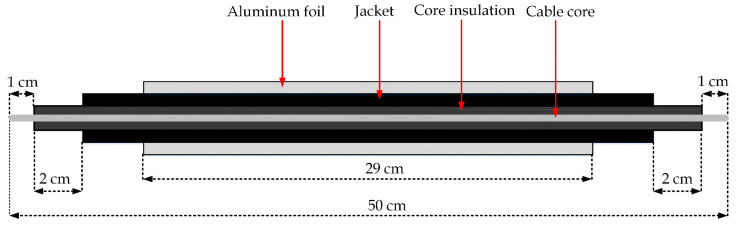
Cross-sectional view of the cable sample.

Moreover, as mentioned, the cable samples under investigation were un-shielded; therefore, the second electrode was provided by wrapping aluminum foil on the test sample’s surface, as shown in Figure 6. To form the foil electrode, 1-cm wide aluminum foil was used, rolled over a length of 29 cm on the cable sheath, with about 50% overlap (Figure 5b). It was placed at the same location on the samples’ surface to avoid any uncertainties. Before starting the aging, all samples were preconditioned for 24 h at 70 °C to remove any moisture within the samples.

## 4. Aging Exposure

The combined radiation-mechanical aging was conducted at the Institute of Isotopes Co., Ltd., Budapest, Hungary. The radiation-mechanical aging was carried out at room temperature using a ^60^ Co gamma-ray source. The distance from the cobalt-60 source corresponds to a specific gamma dose rate. The samples have been exposed to 80 kGy, 160 kGy, 240 kGy, 320 kGy, and 400 kGy with an average dose rate of 0.5 kGy/hr. The dose rate has been selected according to the guidelines of the IAEA [7].

## 5. Methods

### 5.1. Extended Voltage Response (EVR)

**Figure 7:**
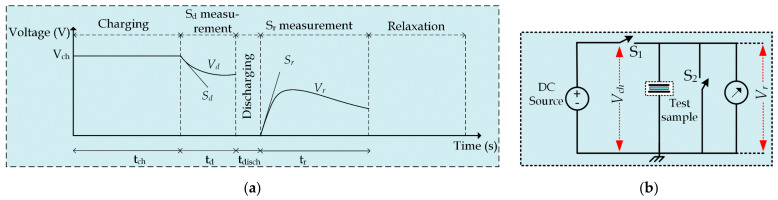
Principle of the VR technique: (**a**) timing diagram; (**b**) circuit illustration.

There are several dielectric condition monitoring techniques, which are used in the condition assessment of electrical insulations. Out of these techniques, the voltage response (VR) technique was presented by Endre Németh in the 1960s [33]. The VR method has been successfully applied to assess the PVC-insulated NPP cables [28]. Moreover, this technique has been adopted in the aging management program of Paks Nuclear Power Plant Ltd., Paks, Hungary, which the Hungarian Atomic Energy Agency has approved. The principle of the VR technique is presented in Figure 7a, while the circuit representation is shown in Figure 7b.

In this technique, by closing the switch *S_1_*, the dielectric material is charged with a DC voltage source (*V_ch_*) for a charging period of (*t_ch_*), as shown in Figure 7a. After the completion of the charging phase, the decay voltage slope (*S_d_*) is measured. The *S_d_* value is proportional to the specific conductivity of the insulation. Then, the test sample is short-circuited by closing switch S_2_ for a discharging time of (*t_disch_*) followed by the return voltage slope (*S_r_*) measurement. The *S_r_* is directly proportional to the polarization conductivity.

**Figure 8:**
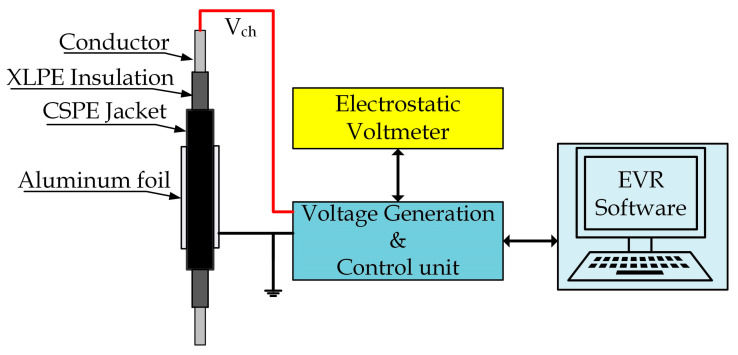
The connection of EVR measurement.

The advanced version of the VR technique, called the extended voltage response (EVR), was used in this research. The EVR technique has been developed at the Budapest University of Technology and Economics. This technique aims to investigate the polarization process over a wide range by measuring the return voltage slopes not only one but multiple discharging times after one charging period [34]. This technique was implemented in many research to assess the condition of NPP cables under different stresses [10,26]. The connection of the EVR measurement is depicted in Figure 8. 

The test parameters of the EVR method were:

- Charging voltage (*V_ch_*): 1 kV;
- Charging time (*t_ch_*): 4000 sec;
- Discharging time (*t_disch_*): from 1 sec (*t_disch1_*) to 2000 sec (*t_disch20_*);
- Number of discharging periods: 20.

### 5.2. Frequency Domain Spectroscopy (FDS)

In the current work, the real (*ε*′) and imaginary (*ε*″) parts of permittivity have been studied over a frequency band of 0.1 Hz to 1 kHz using a dielectric response analyzer type Dirana (OMICRON GmbH, Kiel, Austria). Based on the measured resistance (*R_m_*) and capacitance (*C_m_*) at each particular frequency (*f*), *ε*′ and *ε*″ are calculated as in Equations (1) and (2).

(1) $$\varepsilon^{\prime }\left(f\right)=\frac{C_{m}\left(f\right)}{C_{0}}$$

(2) $$\varepsilon^{\prime \prime }\left(f\right)=\frac{1}{2\pi fC_{0}R_{m}\left(f\right)}$$

The geometric capacitance (*C_0_*) is related to the dimensions of the cable, and it is defined as in Equation (3).

(3) $$C_{0}=\frac{2\pi \varepsilon_{0}l}{\ln\left(\frac{R_{2}}{R_{1}}\right)}$$

(*R*_1_) and (*R*_2_) are denoted as the insulation outer and inner radius. $\varepsilon_{0}$ is the vacuum permittivity, and (*l*) is the aluminum foil length (29 cm).

The Dirana instrument test parameters were:

- General dielectric test type configuration;
- Output voltage: 200 V (peak-peak).

**Figure 9:**
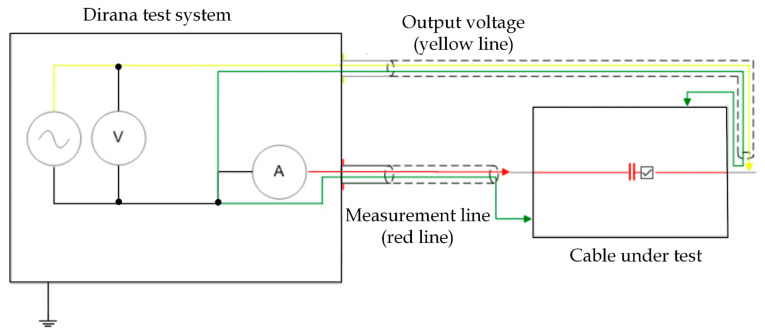
FDS measurement schematic diagram.

The schematic diagram of the FDS measurement on the cable sample is illustrated in Figure 9.

### 5.3. Test Conditions of Electrical Measurements

The following practical considerations have been taken into consideration:

1. Virtually, electromagnetic interferences and noise affect the dielectric measurements; thus, the electrical measurements were carried out while the samples were placed inside a Faraday’s cage;
2. Floating potentials may arise in the case of multi-point grounding; therefore, the whole grounding system has been achieved via single-point grounding;
3. The test temperature was 50 °C (in the oven), and the relative humidity was approximately 10%. These test conditions, i.e., temperature and humidity, help obtain a better dielectric response as the polymers chains and charge carriers’ mobility increase at higher temperatures [7]. Moreover, the role of humidity in the measurement can be ignored.

### 5.4. Shore D Hardness

The change in the mechanical properties of the overall cable insulation was studied by conducting the Shore D hardness test. The hardness test was carried out at ten different points along the cable length, and the average value has been considered in the analysis. The Shore D hardness test was performed using a handheld hardness tester type HPE-II manufactured by Bareiss Prüfgerätebau GmbH, Oberdischingen, Germany.

## 6. Results

For simplicity, all the results are presented as a function of the absorbed dose. However, the cable samples were exposed to parallel radiation-mechanical aging, but the bending stress, i.e., bending radius, was constant during the whole aging period.

### 6.1. Permittivity

**Figure 10:**
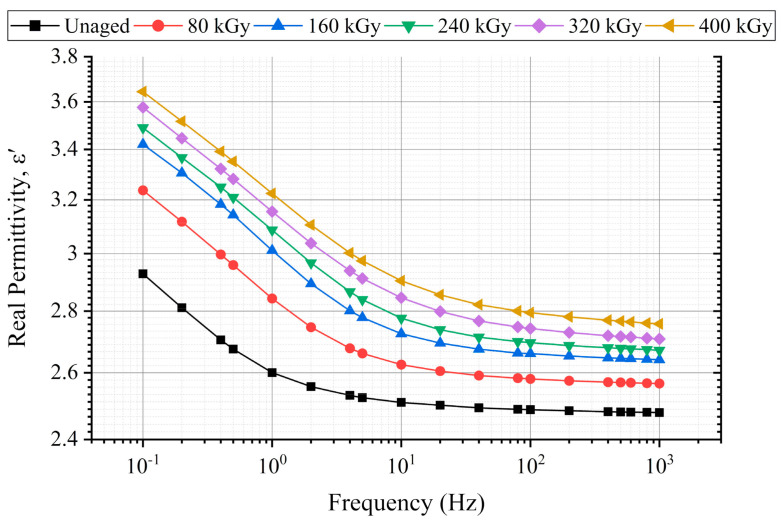
Real permittivity-frequency dependence at different radiation doses.

**Figure 11:**
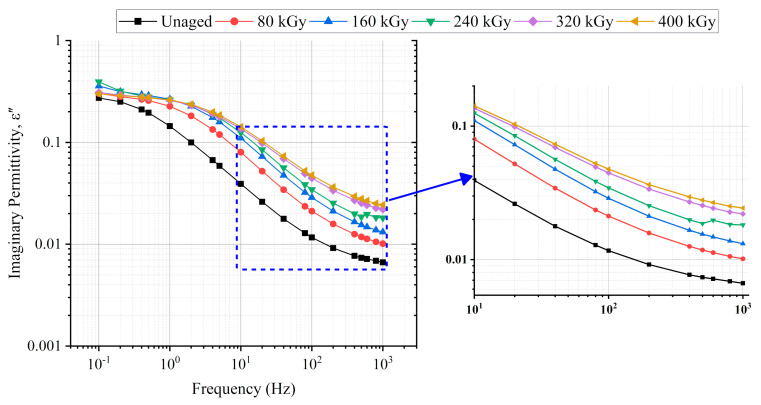
Imaginary permittivity-frequency dependence at different radiation doses (**left**) and the enlarged profile between 10 Hz to 1 kHz (**right**).

The real permittivity behavior at different radiation doses is presented in Figure 10. The real permittivity showed a regular increasing trend with aging. Similarly, the imaginary permittivity versus the frequency is illustrated in Figure 11 (left). The imaginary permittivity slightly changed between 0.1 Hz and 2 Hz. At frequencies higher than 2 Hz, the imaginary permittivity monotonically increased with aging, as shown in the enlarged profile depicted in Figure 11 (right).

### 6.2. EVR

**Figure 12:**
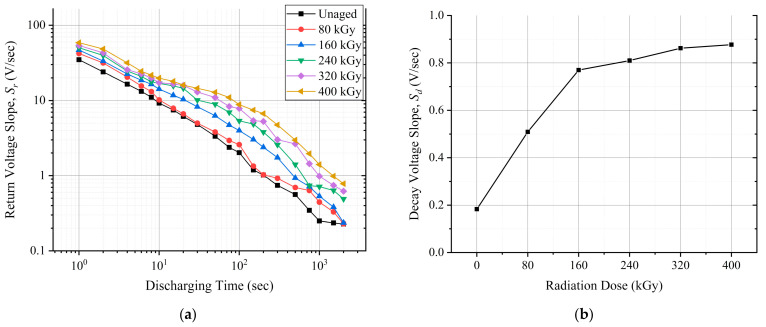
The EVR measurement results: (**a**) return voltage slope versus discharging time; (**b**) decay voltage slope against the absorbed dose.

Figure 12a illustrates the return voltage slope, *S_r_* results as a function of the discharging time after each absorbed dose. The *S_r_* has shifted up with aging. Similarly, the decay voltage slope *S_d_* also increased with aging, as depicted in Figure 12b.

### 6.3. Hardness

**Figure 13:**
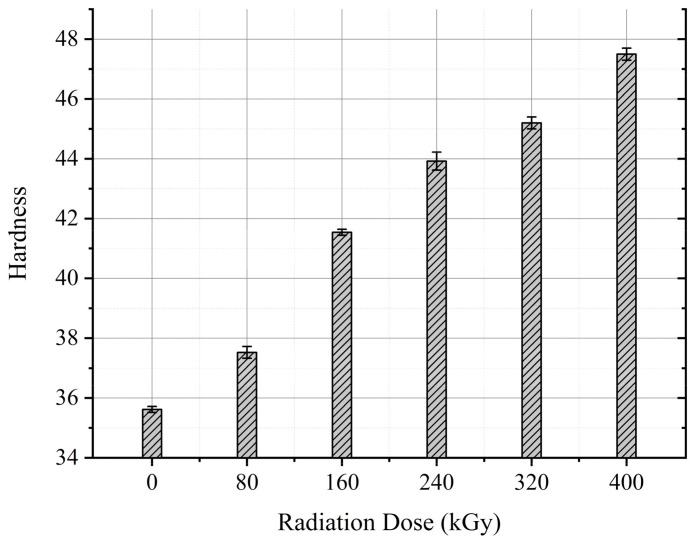
Cable hardness against the absorbed dose.

The mean value of the Shore D hardness measurement after each aging cycle is drawn in Figure 13. It can be noticed that the cable hardness has increased with aging.

## 7. Discussion

### 7.1. Change in Real and Imaginary Parts of Permittivity

**Figure 14:**
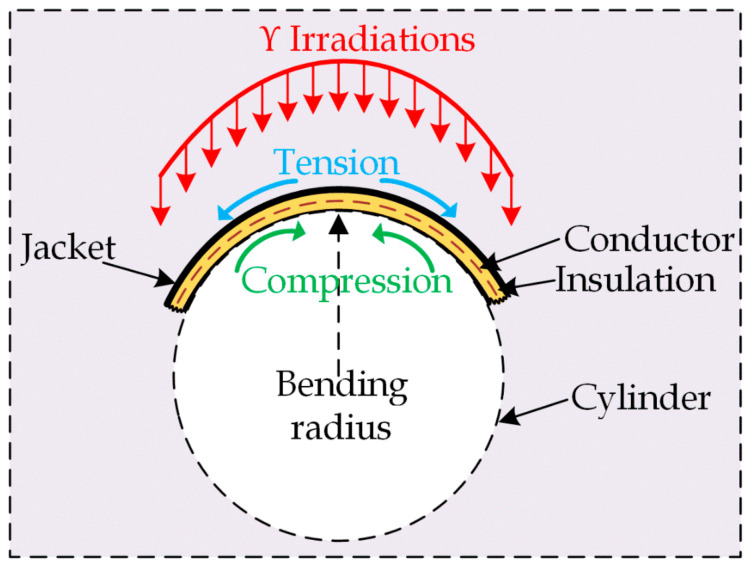
Different stresses were affecting the insulation.

NPP cables are exposed to low doses of γ irradiations during normal operation. Furthermore, they undergo a high level of γ and β irradiations during the accident conditions. As previously mentioned in Section 4, the cable samples under investigation have been subjected to combined radiation-mechanical stresses where different and opposite reactions occurred simultaneously (Figure 14). Thus, molecular structure changes arise due to chemical reactions. Besides the oxidation process, cross-linking, and chain-scission are two opposite reactions that may occur within the same polymer simultaneously [15]. As a result of these reactions, polar molecules and groups are generated, which respond to the external field through orientation.

The real and imaginary parts of permittivity are related to the degree of polarization and the loss of the dielectric material, respectively. The cross-linking reaction forms a three-dimensional network within the polymer matrix, limiting the mobility and orientation of dipoles. Therefore, the cross-linking tends to decrease the insulation’s real permittivity. On the other hand, chain-scission and oxidation reactions result in the generation of small molecules (species) that can orient themselves under the application of an external field. Hence, the real permittivity increases [29].

As illustrated in Figure 14, the mechanical bending stress encompasses two mechanical stresses. The outer insulation section was subjected to tension stress. In contrast, the inner section was exposed to compression stress. The tension stress helps the macromolecules to move easily; also, the molecular chains are stretched along the direction of the tension stress. Thus, the attraction bonds between the molecular chains are fractured, causing stress concentration on the polymer main chain [35]. Compared to the tensile stress, the compressive stress decreases the distance between the molecular chains; thus, more attraction bonds are generated. Besides, the compression stress reduces the free volume due to the compression of the micro-cavities that may arise under the tensile stress [17,36,37,38].

XLPE and CSPE are semi-crystalline polymers where the amorphous and crystalline regions are linked together by tie molecules [39,40]. During radiation and mechanical aging, free radicals are generated in both regions. Radicals formed in the amorphous phase can bond with oxygen or even with each other.

**Figure 15:**
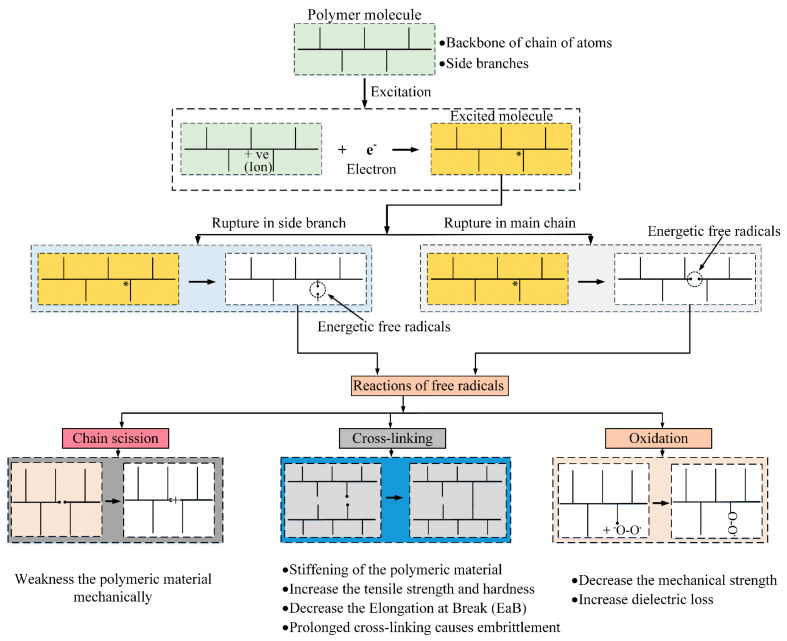
Structural changes due to chemical reactions and their effect.

In contrast, the mobility of free radicals in the crystalline area is limited; thus, they are trapped in the crystalline region, altering the performance of the dielectric material [27]. Furthermore, the trapped radicals tardily migrate to the interface between the amorphous and crystalline phases, leading to bond with oxygen which causes more oxidative degradation, Figure 15 [26,41].

In the amorphous phase, chemical reactions, cross-linking, chain-scission, and oxidation, occur easily. In the presence of oxygen, polar groups are generated as the chain-scission reaction becomes more dominant in the amorphous region. While in the crystalline phase, the molecular chain movement is restricted; thus, the oxidation and cross-linking reactions are limited [30]. The effects of the chain-scission, cross-linking, and oxidation chemical reactions on the polymeric insulation are shown in Figure 15.

**Figure 16:**
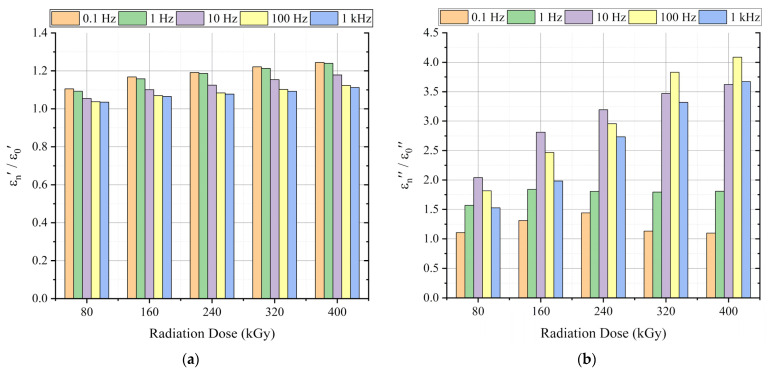
Change in the permittivity to the new case ($\varepsilon_{n}/\varepsilon_{0}$) at 0.1 Hz, 1 Hz, 10 Hz, 100 Hz, and 1 kHz: (**a**) Change in real permittivity; (**b**) change in imaginary permittivity.

Figure 16 presents the change in the real and imaginary parts of permittivity to the new case at 0.1 Hz, 1 Hz, 10 Hz, 100 Hz, and 1 kHz. Compared with 100 Hz and 1 kHz, the change in the real permittivity (Figure 16a) at each absorbed dose was greater at frequencies 0.1 Hz, 1 Hz, and 10 Hz. As in literature, the lowest frequencies are related to the interfacial polarization phenomena, and from tens of Hz, the orientation polarization takes place [2].

Since the whole samples were aged, i.e., neither the core insulation (XLPE) nor the outer jacket (CSPE) of the tested cable were removed during aging, and both polymers contain crystalline and amorphous phases chemical and physical interfaces are also in the cable insulation [2,26,27]. The notable increase in the real permittivity change at 0.1, 1, and 10 Hz is attributed to the dipolar polarization, which is due to the chain-scission dominant reaction, which results in the formation of chemical products such as hydroxyl (O=H) and carbonyl (O=C) groups [42]. It is supported by the change of the imaginary part of permittivity (Figure 16b), which significantly increased at 10 Hz, 100 Hz, and 1 kHz. At the same time, a small change was observed at 0.1 Hz and 1 Hz. The noticeable increase of the imaginary permittivity at 1 kHz suggests an increase in the dipolar polarization due to the presence of the polar groups. 

### 7.2. Central Frequency (CF) and Central Permittivity (CP)

To better understand the real and imaginary parts of permittivity behavior, quantities called central real permittivity (*CRP*), real permittivity’s central frequency (*RPCF*), central imaginary permittivity (*CIP*), and imaginary permittivity’s central frequency (*IPCF*) were deducted, similarly to [43]. The *CRP*, *CIP*, *RPCF*, and *IPCF* are expressed in (4)–(7), respectively.

(4) $$CRP=\frac{\sum_{i=1}^{n}log_{10}f_{i}\cdot \varepsilon_{i}^{\prime }\left(f_{i}\right)}{\sum_{i=1}^{n}log_{10}f_{i}}$$

(5) $$CIP=\frac{\sum_{i=1}^{n}log_{10}f_{i}\cdot \varepsilon_{i}^{\prime \prime }\left(f_{i}\right)}{\sum_{i=1}^{n}log_{10}f_{i}}$$

(6) $$RPCF=10^{\frac{\sum_{i=1}^{n}log_{10}f_{i}\cdot \varepsilon_{i}^{\prime }\left(f_{i}\right)}{\sum_{i=1}^{n}\varepsilon_{i}^{\prime }\left(f_{i}\right)}}$$

(7) $$IPCF=10^{\frac{\sum_{i=1}^{n}log_{10}f_{i}\cdot \varepsilon_{i}^{\prime \prime }\left(f_{i}\right)}{\sum_{i=1}^{n}\varepsilon_{i}^{\prime \prime }\left(f_{i}\right)}}$$

Generally, the *CRP* and *CIP* data represent the variation of the mean polarization peak and the average loss with aging over the studied frequency band [43].

**Figure 17:**
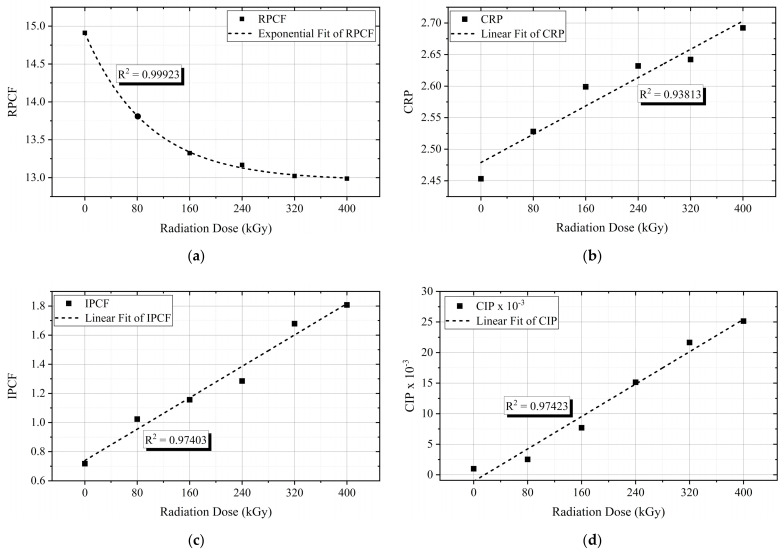
Central permittivity and central frequency profiles: (**a**) real permittivity central frequency (*RPCF*); (**b**) central real permittivity (*CRP*); (**c**) imaginary permittivity central frequency (*IPCF*); (**d**) central imaginary permittivity (*CIP*).

As a function of the irradiation dose, the *RPCF* declined with aging (Figure 17a), while the polarization peak, *CRP*, shifted up with the increase of the irradiation dose (Figure 17b). The *IPCF* (Figure 17c), and *CIP* (Figure 17d), presented an increasing linear trend with each absorbed dose. The shifting of the *IPCF* toward higher frequencies indicates that the main polarization processes in the examined frequency ranges become faster. The increase of the *CIP* means the average loss increases with aging. Nevertheless, these characteristics are very good indicators of aging, owing to the high R^2^ values.

### 7.3. Return and Decay Voltage Slopes

**Figure 18:**
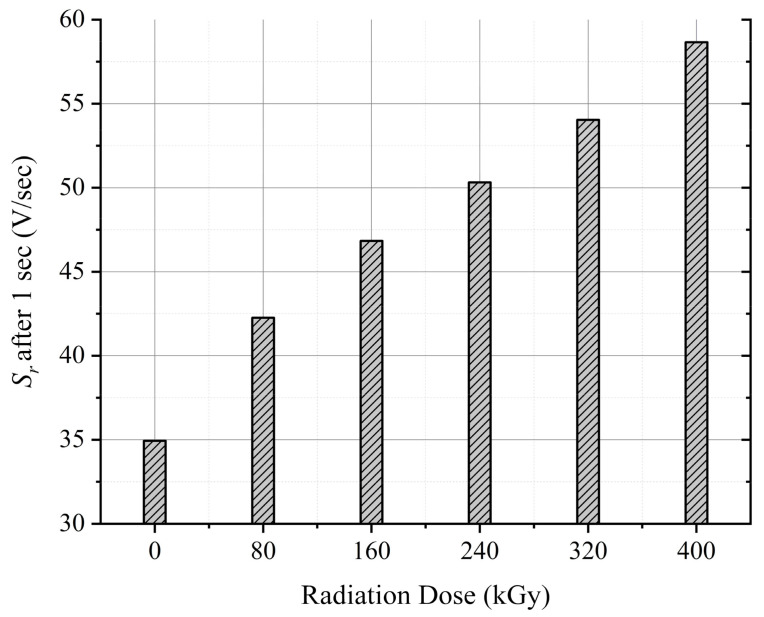
Return voltage slope after 1 sec discharging versus the irradiation dose.

As previously mentioned, the return voltage slope, *S_r_*, is related to the slow polarization processes such as interfacial polarization. The monotonic increase of the *S_r_* values after each aging cycle is also an indicator for the rise of the interfacial polarization. Figure 18 depicts the *S_r_* data after a discharging period of 1 sec to better visualize the *S_r_* trend.

The degradation of the polymer backbone is also evident since the decay voltage slope, *S_d_*, has increased with each aging cycle, as illustrated in Figure 12b. The increasing *S_d_* values are due to the increased dc conductivity of the insulation. The increased conductivity can result from the formation of double bonds in the polymer backbone. The double bonds act as shallow traps enabling charge transportation in the polymer. 

### 7.4. Hardness

Embrittlement is a very important consequence of the degradation of polymers. It has been noticed that the elongation at break of both XLPE and CSPE materials declines with aging [26]. The hardness of XLPE and CSPE as semi-crystalline polymers is related to the stiffness of both crystalline and amorphous phases [26,44]. The increases of the overall cable hardness (see Figure 13) denote the excessive degradation of the XLPE and CSPE materials as the cross-linking reaction along with the chain-scission and oxidation reactions.

## 8. Non-Destructive Aging Indicators

**Figure 19:**
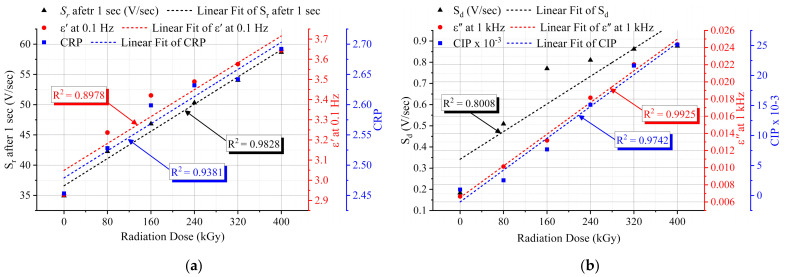
Correlation between the measured electrical parameters and the deducted quantities: (**a**) *S_r_* after 1 sec, real part of permittivity (ε′) at 0.1 Hz, and *CRP*; (**b**) *S_d_*, imaginary part of permittivity (ε″) at 1 kHz, and *CIP*.

**Figure 20:**
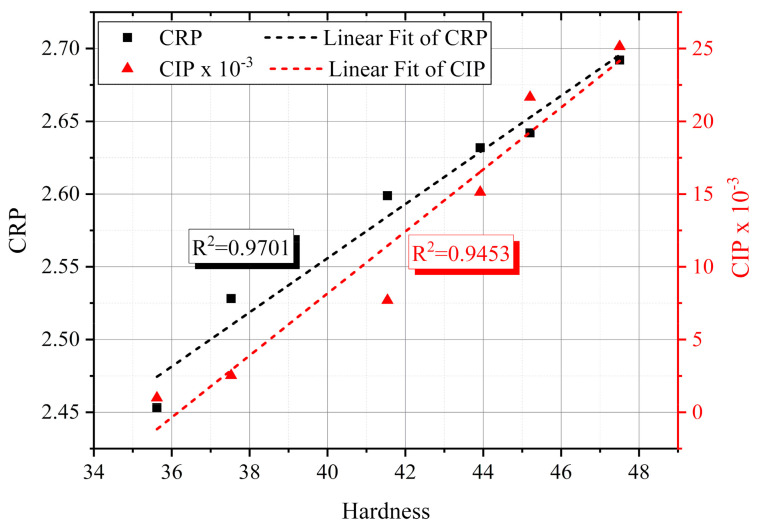
Cross plot of the Shore D hardness, *CRP*, and *CIP*.

Non-destructive aging indicators or markers are essential for the life-extension concern of NPPs since they must have the ability to reveal the degradation level of cable insulation non-destructively. Thus, relationships between the insulation electrical and mechanical parameters were established, and the R^2^ values were considered. As shown in Figure 19a, the return voltage slope, *S_r_*, presented a good agreement with the real permittivity at 0.1 Hz and the *CRP*. Furthermore, the decay voltage slope, *S_d_*, introduced a sturdy matching with the behavior of the imaginary permittivity at 1 kHz and the *CIP* (Figure 19b). Considering the Shore D hardness behavior, the *CRP* and *CIP* were strongly correlated with the hardness data (Figure 20). The high R^2^ values, Figure 19 and Figure 20 show the applicability of the implemented non-destructive techniques to assess the insulation degradation of the NPP LV cables, at least the cable samples under investigation. 

## 9. Conclusions

This work aimed to implement non-destructive condition monitoring techniques to monitor the impact of simultaneous radiation-mechanical aging on the insulation of XLPE/CSPE unshielded LV NPP power cable samples. To achieve this goal, the real and imaginary parts of permittivity, the decay and return voltage slopes, and the Shore D hardness techniques were conducted. A monotonic increase in the real part of permittivity was observed with aging. Moreover, the imaginary part of permittivity increased with aging at frequencies between 10 Hz and 1 kHz. Moreover, the return and decay voltage slopes increased with each absorbed dose, and the aging increased the hardness of the cable’s overall insulation.

Moreover, new quantities, *CRP*, *CIP*, *RPCF*, and *IPCF* were deducted based on the permittivity data and the frequency range under consideration (0.1 Hz to 1 kHz).

The low-frequency real permittivity at 0.1 Hz and the *CRP* have presented a strong correlation with the return voltage slope, *S_r_* after 1 sec. Likewise, the imaginary permittivity at 1 kHz and the *CIP* were matched with the decay voltage slope. Similarly, the deducted quantities, *CRP*, and *CIP* have also agreed with the hardness trend.

Therefore, the implemented electrical condition monitoring techniques and the deducted quantities can be used as non-destructive aging indicators to investigate the condition of cable polymeric insulation used in NPPs. The condition assessment of cable insulation-based electrical measurements, unlike mechanical testing, is not a non-destructive diagnostic technique. It can also be conducted in situ; hence, the NPP cables can be safely and effectively monitored.

## Figures and Tables

**Table 1:** Cable technical data.

Parameter	Value
Cable type	Single-core unshielded
Nominal voltage (kV)	0.6
Conductor size (AWG)	6
Number of strands	7
Core insulation	XLPE
Insulation thickness (Mils)	45
Jacket material	CSPE
Jacket thickness (Mils)	30
Overall diameter (inch)	0.34
Bend radius permanent training (inch)	1.5
Bend radius during installation (inch)	2.75
Max. conductor temperature (°C)	120

The data given in Table 1 are as per the cable datasheet.

## Data Availability

The data presented in this study are available on request from the corresponding author.

## Abbreviations

NPP	Nuclear power plant
LOCA	Loss of coolant accident
I&C	Instrumentation and control
LV	Low-voltage
MV	Medium-voltage
XLPE	Cross-linked polyethylene
EPR	Ethylene-propylene rubber
CSPE	Chlorosulfonated polyethylene
EaB	Elongation at break
EVR	Extended voltage response
VR	Voltage response
PVC	Polyvinyl chloride
*S_d_*	Decay voltage slope
*S_r_*	Return voltage slope
ε′	Real part of permittivity
ε″	Imaginary part of permittivity
CF	Central frequency
CP	Central permittivity
CRP	Central real permittivity
RPCF	Real permittivity central frequency
CIP	Central imaginary permittivity
IPCF	Imaginary permittivity central frequency
